# Cancer Treatment Response to Checkpoint Inhibitors Is Associated with Cytomegalovirus Infection

**DOI:** 10.7759/cureus.6670

**Published:** 2020-01-15

**Authors:** Faith Villanueva, Cai Yuan, Walter Drane, Long Dang, Thu-Cuc Nguyen

**Affiliations:** 1 Internal Medicine, University of Florida, Gainesville, USA; 2 Oncology, University of Florida, Gainesville, USA; 3 Radiology, University of Florida, Gainesville, USA; 4 Oncology, Ochsner Health System, Baton Rouge, USA; 5 Internal Medicine, Moffitt Cancer Center, Tampa, USA

**Keywords:** immune therapy, cmv infection, urothelial carcinoma, pd-1 inhibitor, malignancy, oncology

## Abstract

Programmed cell death protein-1 (PD-1) and programmed cell death-ligand 1 (PD-L1) checkpoint inhibitors induce tumor response by activating the patient’s own immune system to fight cancer. Tumors with high tumor mutational burden or those that express high levels of PD-1/PD-L1 are more responsive to PD1/PDL1 inhibitors. There is much interest in determining how to improve response to PD-1/PD-L1 inhibitors. We report a case of a patient with metastatic bladder cancer who was primarily resistant to treatment with PD-1/PD-L1 inhibitors, then had a complete response after developing cytomegalovirus infection.

## Introduction

The development of immune checkpoint inhibitors is a breakthrough in the field of human cancer research and treatments. Tumor cells evade host immunosurveillance through various mechanisms, including activation of checkpoint pathways that suppress the antitumor effects from the host. Immune checkpoint inhibitors, such as programmed cell death protein-1 (PD-1) and programmed cell death-ligand 1 (PD-L1) inhibitors, exhibit significant antitumor activity and induce durable disease control by restoring an efficient antitumor response [1,2]. Thus, it has become a standard of care for a wide variety of malignancies, including melanoma, renal cell carcinoma, urothelial cancer, lung cancer, and Hodgkin’s lymphoma [3-5]. Complete responses have been achieved in many advance cancers including urothelial cancer [6]. Despite this exciting advance in immune-oncology, it is also recognized that not all cancer patients respond to immunotherapy as the overall response rate of single agent of PD-1/PD-L1 inhibitors in solid tumor remains 20%-40% [7,8]. Therefore, how to improve response to PD-1/PD-L1 inhibitors has been a great interest among bench researchers and clinicians.

While immunotherapies are now widely available to patients, clinicians face a major challenge in determining the efficacy of these novel agents [9]. Pseudoprogression has been recognized as a unique phenomenon when evaluating patients treated with PD-1/PD-L1 inhibitors. Its occurrence was initially noted in the treatment of melanoma using cytotoxic T-lymphocyte antigen-4 inhibitor, ipilimumab [10]. Pseudoprogression has been subsequently reported in the studies of PD-1/PD-L1 inhibitors in various solid tumors [11-14]. It is not a true disease progression, but rather radiographic growth of tumor lesions or appearance of new lesions, which subsequently reduce in tumor burden with continuous treatments [9,14]. As such, the immune-related response criteria (iRECIST) has been introduced as standardized evaluation criteria for this unconventional response patterns with immunotherapeutic agents [15,16]. Usage of traditional response evaluation criteria for solid tumor (RECIST) may result in tumor response misclassification [15].

We report a case of a patient with metastatic bladder cancer who was primarily resistant to treatment with PD-1/PD-L1 inhibitors, then had a complete response after developing cytomegalovirus (CMV) infection.

## Case presentation

**Figure 1:**
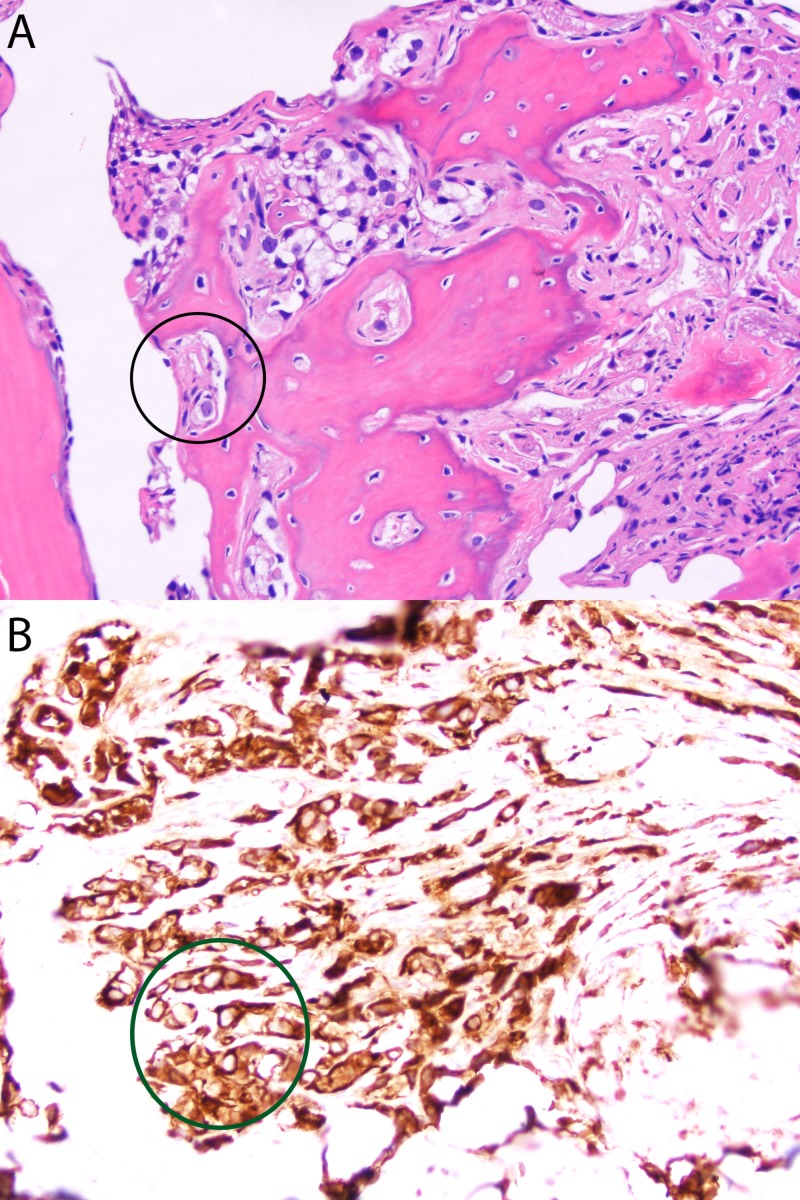
Left iliac crest biopsy Histology of left iliac crest biopsy revealed epithelioid malignant cells infiltrating the bone (A, H&E stain) which are confirmed to be cytokeratin positive (B, immunostain for AE1/AE3). The tumor cells were also positive for cytokeratin 7 and p40, but negative for CK20. The histomorphology and immunophenotype confirmed the diagnosis of metastatic urothelial carcinoma. Key: black circle, epithelioid malignant cells; green circle, highlighted tumor cells positive for cytokeratin 7.

A 67-year-old woman presents with a history of high-grade urothelial carcinoma diagnosed on transurethral resection of bladder tumor (TURBT) during workup for gross hematuria. She has a distant history of colorectal cancer that was successfully treated with right hemicolectomy and two rounds of adjuvant chemotherapy. At the time of diagnosis of urothelial carcinoma, computed tomography (CT) of the abdomen and pelvis did not show evidence of metastatic disease, and she subsequently underwent neoadjuvant chemotherapy with four cycles of cisplatin/gemcitabine, followed by radial cystectomy. Bladder pathology showed pT2 disease with negative lymph nodes and margins. However, 22 months after diagnosis, a positron emission tomography (PET)-CT scan showed widespread progression of disease involving pelvic/para-aortic lymph node and extensive bony metastases. The PD-L1 expression was not evaluated; however, after discussion with patient, immunotherapy was chosen as she declined chemotherapy due to significant side effects from prior adjuvant chemotherapy for her colon cancer. She was subsequently started on atezolizumab and underwent stereotactic body radiation therapy to the left femoral neck. Left iliac crest biopsy (Figure 1) was consistent with metastatic urothelial carcinoma.

**Figure 2:**
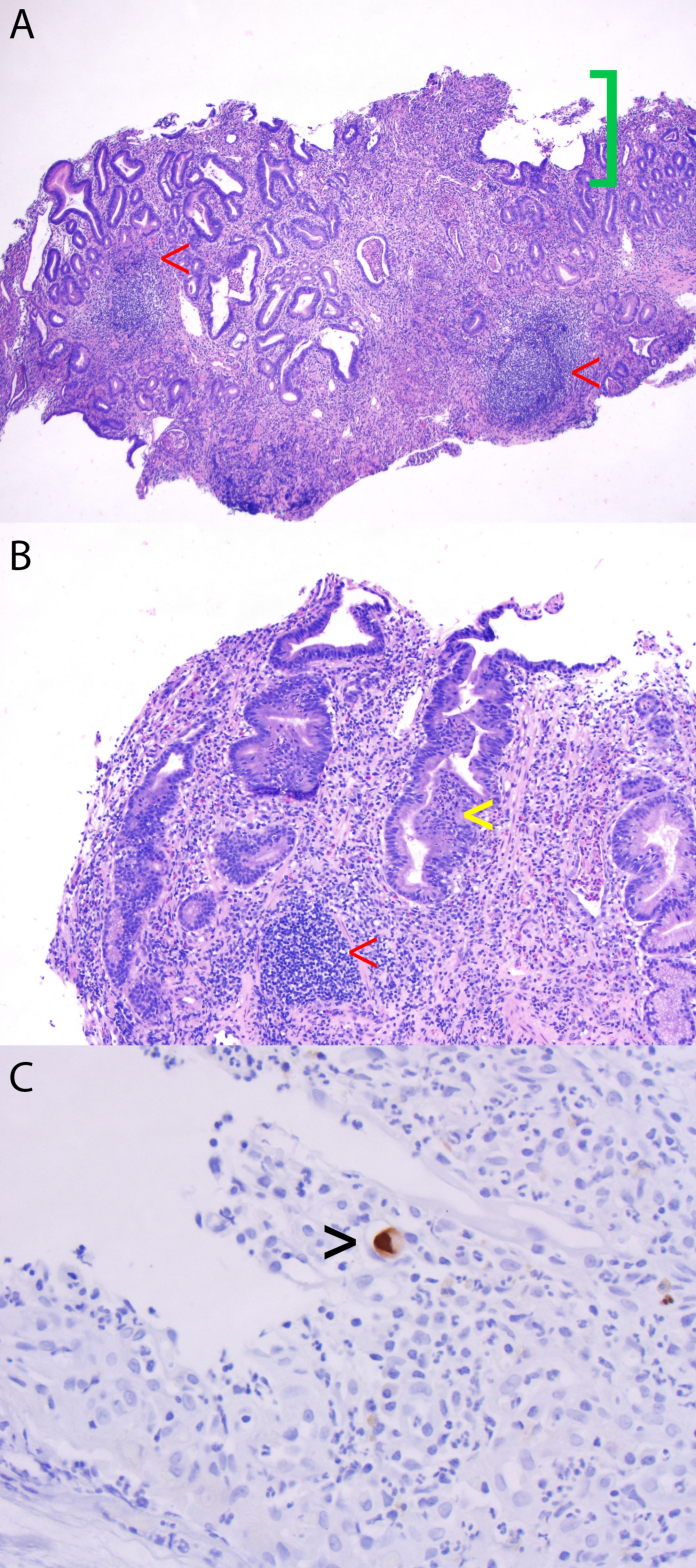
Gastric antrum and body biopsies Histology of gastric antrum and body biopsies revealed severe chronic active gastritis with ulceration. The antrum has dense lymphoplasmacytic inflammation, neutrophilic inflammation in the lamina propria and glands, intraepithelial lymphocytosis, and crypt apoptosis and necrosis (A, H&E stain). The body biopsy shows atrophy of oxyntic glands, dense lymphoplasmacytic infiltrate, neutrophilic inflammation in the lamina propria and glands (B, H&E stain). Immunohistochemistry for cytomegaloviral antigen performed on the antral biopsy revealed several cytomegalovirus (CMV) inclusions (C, immunostain). Key: red arrow, dense lymphoplasmacytic inflammation and infiltrate; green bracket, ulceration; yellow arrow, atrophy of oxyntic glands; black arrow, CMV inclusion.

Repeat PET-CT scan after six months of atezolizumab showed progression of osseous metastatic disease, and she was switched to pembrolizumab. Her disease continued to progress radiographically while on immune therapy. After nine months of immune therapy, she experienced progressive, intractable epigastric pain, and she was found to have CMV gastritis confirmed on gastric antral and body biopsy (Figure 2) obtained during esophagogastroduodenoscopy (EGD). Grossly, her EGD showed diffuse severely erythematous mucosa with bleeding on contact was found in the entire examined stomach. At the time of diagnosis, her serum CMV titers were detected, but less than 100 copies/mL.

**Figure 3:**
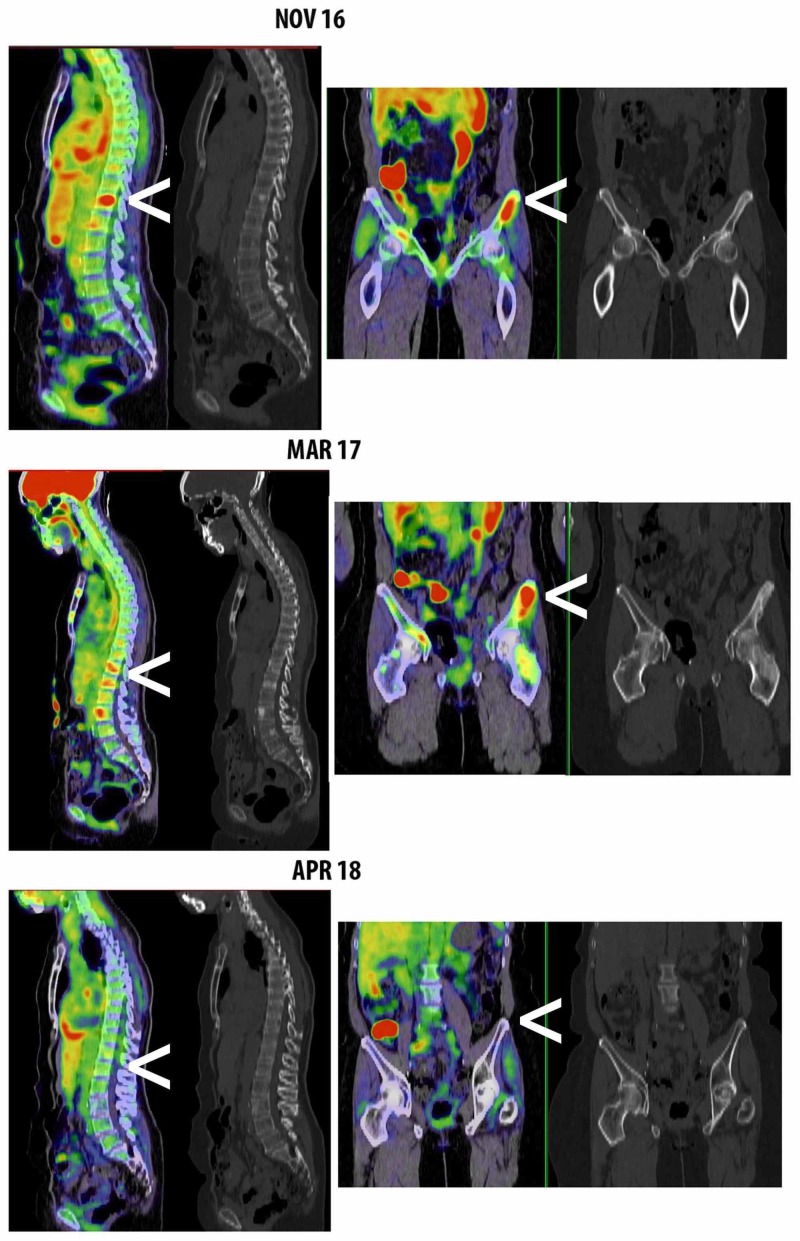
Serial PET-CT imaging Sagittal and coronal views of positron emission tomography-computed tomography (PET-CT) show initial progression of disease (from November 2016 to March 2017) and subsequent response (from March 2017 to April 2018) to immune therapy after infection and treatment of cytomegalovirus (CMV) with select areas indicated by white arrows.

**Figure 4:**
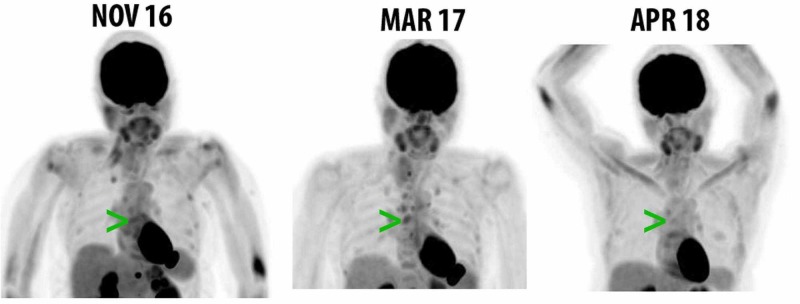
Serial maximum intensity projection visualization Coronal views of maximum intensity projection visualization showing initial progression of disease (from November 2016 to March 2017) and subsequent response (from March 2017 to April 2018) to immune therapy after infection and treatment of cytomegalovirus (CMV). Key: green arrows, avid lesion in the anterior costal junction of the left sixth rib that progresses from November 2016 to March 2017, with regression on April 2018 image.

She was treated with intravenous ganciclovir with complete resolution of gastritis symptoms. Immune therapy was held as she recovered from the CMV gastritis. After a three-month immune therapy hiatus, she resumed treatment with pembrolizumab without further complication. Follow-up PET-CT after four and 12 months showed a sequential decrease in fluorodeoxyglucose (FDG) uptake in multiple lymph nodes and bony metastases, consistent with radiographic improvement in disease approaching complete remission (Figures 3, 4). She also underwent a repeat EGD which showed normal gastric mucosa, with biopsies negative for CMV inclusions.

While her metastatic urothelial carcinoma initially progressed on immune therapy, since her episode of CMV gastritis, her malignancy showed marked radiographic improvement in nodal and bony metastatic disease with complete remission, and she has remained asymptomatic and is able to perform all activities of daily living independently. She has now completed her last cycle of immune therapy with pembrolizumab, and is undergoing surveillance with serial CT scans with close monthly follow-up with medical oncology.

## Discussion

CMV is a ubiquitous herpes virus that is estimated to affect 40% in industrialized areas and up to 100% in developing areas [17]. Initial infection in immunocompetent host ranges from asymptomatic to febrile, flu-like symptoms, and rarely, can be a systemic syndrome affecting many organ systems. After initial infection, CMV establishes a latent infection and persists in myeloid cells and escapes the host immune system [18]. Latent infection can reactivate with viral replication, causing systemic viral syndrome due to viral replication in the peripheral blood or invasive disease with symptoms at the affected organ. In immunocompromised host, e.g. post-transplant setting or HIV infection, CMV infection, whether due to initial infection or viral reactivation, can be severe resulting in end organ damage and even death [18].

Our patient presented with active CMV gastritis after one year of treatment with PD-1/PD-L1 inhibitors. Her cancer had shown true progression prior to developing CMV gastritis. After recovery from CMV gastritis, the patient was resumed on PD-1 inhibitor for treatment of her bladder cancer, and subsequently went into complete remission. Based on the temporal association of CMV gastritis with the subsequent clinical response to PD-1 inhibitor, it is possible that the patient’s immune system is primed by CMV to cause an immune response to her bladder cancer. Our observation is consistent with published research that CMV infection can induce a potent cellular and cytokine-mediated immune response to non-CMV targets [19,20].

## Conclusions

Our case observation is the first to show a link between CMV infection and response to PD-1/PDL-1 inhibitors for cancer treatment. Factors that impact efficacy in immune therapy remain unclear. Our next step is to evaluate large databases for association between CMV infection and cancer outcome with PD-1/PD-L1 inhibitors.
